# Paxillin: A Hub for Mechano-Transduction from the β3 Integrin-Talin-Kindlin Axis

**DOI:** 10.3389/fcell.2022.852016

**Published:** 2022-04-05

**Authors:** Marta Ripamonti, Bernhard Wehrle-Haller, Ivan de Curtis

**Affiliations:** ^1^ Division of Neuroscience, San Raffaele Scientific Institute and Vita-Salute San Raffaele University, Milano, Italy; ^2^ Department of Cell Physiology and Metabolism, University of Geneva, Centre Médical Universitaire, Geneva, Switzerland

**Keywords:** mechano-sensing, tensional force, lim domain, integrin activation, plasma membrane

## Abstract

Focal adhesions are specialized integrin-dependent adhesion complexes, which ensure cell anchoring to the extracellular matrix. Focal adhesions also function as mechano-signaling platforms by perceiving and integrating diverse physical and (bio)chemical cues of their microenvironment, and by transducing them into intracellular signaling for the control of cell behavior. The fundamental biological mechanism of creating intracellular signaling in response to changes in tensional forces appears to be tightly linked to paxillin recruitment and binding to focal adhesions. Interestingly, the tension-dependent nature of the paxillin binding to adhesions, combined with its scaffolding function, suggests a major role of this protein in integrating multiple signals from the microenvironment, and accordingly activating diverse molecular responses. This minireview offers an overview of the molecular bases of the mechano-sensitivity and mechano-signaling capacity of core focal adhesion proteins, and highlights the role of paxillin as a key component of the mechano-transducing machinery based on the interaction of cells to substrates activating the *β*3 integrin-talin1-kindlin.

## Introduction

Focal adhesions (FAs) are specialized integrin-dependent adhesion complexes, which mediate cell anchoring to the extracellular matrix (ECM) (Winograd-Katz et al., 2014). FAs also function as mechano-transducing machineries perceiving and integrating diverse physical and (bio)chemical environmental cues, and transducing them into intracellular signaling pathways (Zaidel-Bar et al., 2007a; Wehrle-Haller, 2012; Yu et al., 2012). Indeed, FAs control cellular programs as diverse as cell adhesion, migration, survival, growth, proliferation, and differentiation (Wehrle-Haller, 2012; Winograd-Katz et al., 2014). To accomplish these diverse regulatory functions, *αβ* heterodimeric integrin receptors (Hynes, 2002) cluster in the plasma membrane, recruit numerous proteins to their cytoplasmic tails, and give rise to a highly dynamic intracellular protein network which has been termed the “integrin adhesome” (Zaidel-Bar et al., 2007a; Winograd-Katz et al., 2014). The tight regulation of its protein composition ensures FAs functioning as mechanical anchoring points, as well as signaling platforms (Wozniak et al., 2004; Zaidel-Bar et al., 2007a; Geiger and Yamada, 2011; Winograd-Katz et al., 2014).

The extensive implication of integrins and FAs-dependent signaling in pathological conditions (Bachmann et al., 2019) pushes current research towards a better understanding of their functioning and spatiotemporal regulation (Wu et al., 2019; Su et al., 2020). A few years ago, interferometric photoactivated localization microscopy (iPALM) has revealed a layered organization of integrin-containing FAs (Kanchanawong et al., 2010; Case et al., 2015). This model, proposing the spatial segregation of specific adhesome components between a integrin signaling layer (closest to the membrane), a force transduction layer, and an actin regulatory layer (innermost), has been endorsed by studies making advantage of diverse techniques, such as single protein tracking microscopy, superresolution microscopy, proximity biotinylation, and bimolecular fluorescence complementation (Dong et al., 2016; Chastney et al., 2020; Legerstee and Houtsmuller, 2021; Orre et al., 2021; Ripamonti et al., 2021). Despite these great advances, the characterization of several structural and mechanical aspects of the sophisticated integrin-dependent protein network, a comprehensive understanding of the FA machinery is still far from being accomplished (Chastney et al., 2020; Legerstee and Houtsmuller, 2021). The decoding of how specific cellular responses can be provoked by a given physiological, pathological, or pharmacological stimulus is challenged by the interdependency of FA players, regulatory systems, including the plasma membrane and its composition, and the tension across integrin receptors (Vogel, 2006; Gauthier and Roca-Cusachs, 2018). In addition, a wide range of post-translational modifications and the expression of many FA protein splice variants and isoforms generate additional layers of complexity that need to be understood to identify specific *versus* more general functions of FAs (Anthis et al., 2010; Choi et al., 2011; Soto-Ribeiro et al., 2019).

**FIGURE 1:**
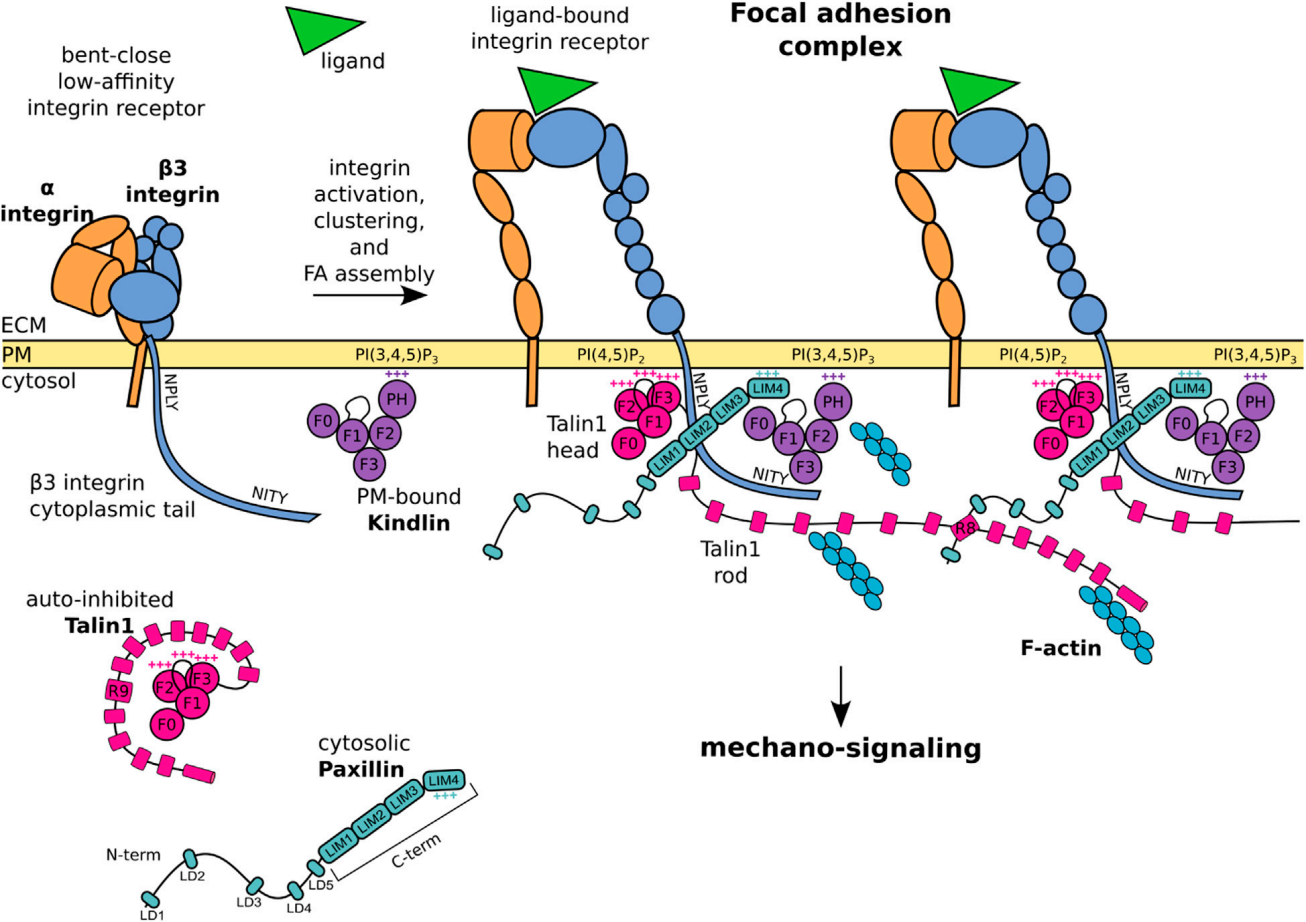
Schematic representation of core FA proteins and their interactions. The ***β*3 integrin subunit** is composed of an extracellular domain, a transmembrane domain, and a C-terminal cytoplasmic tail presenting the membrane proximal NPLY talin-binding and membrane distal NITY kindlin-binding sites. The heterodimeric integrin receptor in a bent-close low-affinity conformation (left) switches to an extended-open high-affinity state and binds ligands and intracellular proteins (right). **Talin1** consists of a globular head, an unstructured linker, and a C-terminal rod domain which intramolecularly interacts with the head domain to keep cytosolic talin1 auto-inhibited. Upon integrin activation, the talin1 F2 domain binds to membrane phospholipid PI(4,5)P_2_, the talin F3 subdomain to the membrane proximal NPLY motif in the β3 integrin cytoplasmic tail, and the talin rod engages the F-actin network. **Kindlin** is similarly organized to talin-head but with the addition of a PH domain inserted within the F2 domain which recognizes membrane phosphoinositides, while the F3 domain binds to the membrane-distal NITY motif in the *β*3 integrin cytoplasmic tail. The **paxillin** amino-terminal half presents five short LD motifs and is followed by the carboxyl-terminal half composed of four LIM domains. The paxillin N-terminal LD1 and LD2 interact with the talin1 R8 domain, the LIM domains point towards the membrane proximal region, the positively charged LIM4 domain interacts with kindlin and the plasma membrane. One of the paxillin LIM domain could recognize the Y presented by the NPLY motif. +++ indicates positively charged regions. For representative purposes, the *β*3 integrin tail is outsized and some protein domains are simplified or omitted for clarity.

**FIGURE 2:**
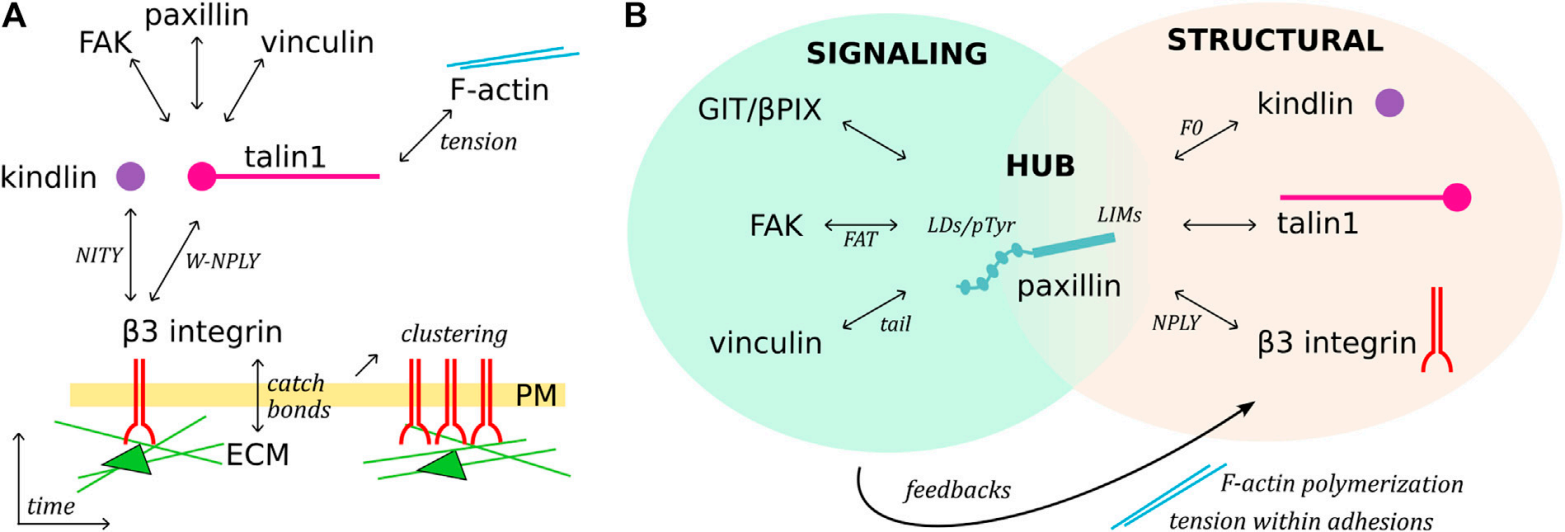
Temporal sequence of *β*3 integrin activation and paxillin-mediated organization of adhesions. **(A)**, Schematic representation of FA assembly over time. Ligand, talin and kindlin binding to integrin receptors triggers their clustering, mediates the mechanical connection with the F-actin network, and recruits cytoplasmic proteins. **(B)**, Paxillin is a structural, signaling and linker component of FAs. C-terminal LIM domains target paxillin to FAs, possibly directly interacting with talin, kindlin, and β3 integrin (putative domains indicated). The paxillin N-terminus functions as a signaling molecule, binding/recruiting different subsets of FA proteins, modulating F-actin polymerization and tension within adhesions, and therefore generating feedback signaling which can lead to FA turnover.

This review offers an overview of the molecular basis of the mechano-sensitivity and mechano-signaling capacity of the core FA proteins β3 integrin, talin1, and kindlin (Figure 1) that enable mechano-transduction. The focus is on *β*3 integrins as a paradigm for paxillin- and mechano-dependent mechanisms that may be extended to other classes of integrins. We will first address *β*3 integrin receptors, their link to talin1 and kindlin, and how paxillin is recruited to this complex for further mechanical stabilization, as well as to elicit diverse signaling pathways. We will highlight the role of paxillin and its central position to integrate the structural changes of the *β*3 integrin-talin1-kindlin complex, and offer evidence that paxillin is not only a scaffold or signaling protein as previously described, but also a key component of the mechano-transducing machinery (Figure 2).

## The *α*v*β*3 Integrin Receptor

Integrins come in different flavors, ranging from diversity in ligand binding and exhibiting specific structural features (Hynes, 2002; Bachmann et al., 2019), which makes it impossible to cover the entire family in this review. For historical reasons the *α*v*β*3 integrin is one of the best studied receptors, with implication in many pathophysiological settings (Zhu C. et al., 2019), representing a typical example of many integrin-dependent functions.

The *β*3 integrin receptors comprise two heterodimers originating from the pairing of the *β*3 subunit with either αIIb or αv chains, creating the *α*IIb*β*3 and the *α*v*β*3 heterodimers, respectively (Hynes, 2002; Bachmann et al., 2019). The αIIbβ3 integrin complex is a platelet-specific receptor which is activated by multiple signaling cascades to trigger platelet activation and aggregation (Ye et al., 2012; Huang et al., 2019). In contrast, the αvβ3 integrin has a wider expression and physiological functions related to tissue repair and inflammation in osteoclasts, platelets, megakaryocytes, kidney, vascular smooth muscles, endothelium, and placenta (Horton, 1997). In addition, αvβ3 is upregulated in endothelial cells undergoing tumor-induced angiogenesis (Mahabeleshwar et al., 2007), as well as in many tumor cells (Horton, 1997; Vonlaufen et al., 2001). The αvβ3 integrin recognizes the Arg-Gly-Asp (RGD)-tripeptide-containing sequence present in different ECM ligands (Pytela et al., 1985; Horton, 1997; Humphries et al., 2006), and preferentially binds to vitronectin and osteopontin, especially under low force conditions (Bachmann et al., 2020). Changes in mechanical cues of the microenvironment enlarge the ligand preference of αvβ3 integrin, and can induce mis-regulation of integrin-dependent signaling pathways (Bachmann et al., 2020), as in the case of pathological ECM stiffening in the tumor niche (Attieh et al., 2017). The αvβ3 receptor also plays a role in tumor progression and metastasis formation: by controlling the actin cytoskeleton (Havaki et al., 2007); by supporting tumor cell binding to, and transmigration across activated endothelia (Saalbach et al., 2005); by synergizing with VEGF-dependent pathways to promote angiogenesis (Mahabeleshwar et al., 2007); and by sustaining the activation of the Src kinase (Huveneers et al., 2007). Importantly, αvβ3 FAs localize proteolytically active matrix metalloproteinases at the cell surface (Brooks et al., 1996) and support efficient directed cell migration to promote metastasis formation (Ballestrem et al., 2001).

## The *β*3 Integrin-talin1-Kindlin-Paxillin Complex

The assembly of FAs requires the conformational switch of the β3 integrin receptor from a bent-close low-affinity, to an extended-open high-affinity state (Figure 1). This activation can be triggered by the binding of the intracellular adapter proteins talin1 and kindlin to the cytoplasmic tail of the β3 integrin (Hytonen and Wehrle-Haller, 2014; Bachmann et al., 2019; Huang et al., 2019). These integrin activators are essential for integrin-dependent attachment and spreading: talin-null cells and kindlin-null cells display a non-adherent phenotype (Bottcher et al., 2017), suggesting a lack of transmission of mechanical signals and of cellular responses.

The progression from first integrin-adapter interactions and ECM-ligand binding toward FA maturation involves integrins clustering and their mechanical connection to the intracellular actin network (Thievessen et al., 2013; Hytonen and Wehrle-Haller, 2014) (Figure 1; Figure 2A). During this process, force is a key player acting at several steps. For example, catch bonds (*i.e.* force-dependent bonds strengthened by the force applied along the receptor) are formed at the level of the integrin-ligand interaction (Gauthier and Roca-Cusachs, 2018), while the mechanical tension along the integrin-adapters-actin axis leads to the exposure of cryptic binding sites in the talin C-terminal rod domain (Del Rio et al., 2009; Rahikainen et al., 2017), thus favoring the interaction with adapter proteins (e.g., paxillin, vinculin, and FAK) and the assembly of multiprotein signaling complexes (Hytonen and Vogel, 2008; Hytonen and Wehrle-Haller, 2014; Goult et al., 2018).

### Talin1

By virtue of its structure, talin fulfils the role of a mechano-sensor of the extracellular rigidity, as well as of a mechano-transducer (Austen et al., 2015; Gough and Goult, 2018). The talin N-terminal head domain binds to integrins, induces conformational changes of the juxtamembrane- and ecto-domains of the integrin receptor, and stimulates integrin activation and clustering (Wegener et al., 2008; Saltel et al., 2009). Alongside, the binding of the talin C-terminal rod domain to the F-actin network directly transmits mechanical forces to the cellular cytoskeleton (Zhang X. et al., 2008; Rahikainen et al., 2017). As a feedback mechanism, the stretching of the talin rod domain reveals additional binding sites and ensures a tension-dependent recruitment of cytoplasmic proteins to the adhesion complexes (Figure 1; Figure 2A).

The talin N-terminal head domain consists of a globular FERM domain (F0 to F3 subdomains) (Zhang et al., 2020), connected by an unstructured linker to a C-terminal rod domain, which contains 13 *α*-helical bundles (R1 to R13) (Rahikainen et al., 2017) (Figure 1). The interaction of the talin head with the talin rod domain (*via* F3-R9) keeps cytosolic talin in a globular, autoinhibited conformation (Goksoy et al., 2008; Calderwood et al., 2013) (Figure 1). The recruitment of the talin head to the plasma membrane is controlled by two mechanisms: 1) the binding of the membrane-bound Rap1 GTPase to the F0 and F1-subdomains (Lagarrigue et al., 2020), and 2) the simultaneous binding of the F1-loop and the F2 subdomains to the membrane phospholipid PI(4,5)P_2_ (Anthis et al., 2009; Saltel et al., 2009; Goult et al., 2010). The concomitant membrane association of a basic loop in the talin F3 subdomain leads to the release of the autoinhibition, the detachment of the C-terminal domain, and the exposure of an additional *β*3 integrin tail binding site in the talin F3 subdomain, which assures binding to a juxtamembrane acidic motif, as well as the membrane proximal W-NPLY peptide, in the β3 integrin cytoplasmic tail (Cluzel et al., 2005; Wegener et al., 2008; Saltel et al., 2009; Zhang et al., 2020) (Figure 1). Full integrin activation and clustering however requires an F1-loop mediated interaction with the inner-membrane clasp to open the inhibitory salt-bridge formed between the αv and β3 integrin tails (Kukkurainen et al., 2020; Lagarrigue et al., 2020).

Once the auto-inhibited conformation of talin is released, talin engages the F-actin network: either directly through the two main actin-binding sites in the rod domain (Figure 1; Figure 2A); or indirectly, through the interaction with F-actin-bound vinculin (Humphries et al., 2007; Austen et al., 2015; Rahikainen et al., 2017; Gough and Goult, 2018; Atherton et al., 2020). When this mechanical connection is established, the application of tension results in the reversible unfolding of the talin rod, which reveals cryptic binding sites and allows the conversion of the tensional force on the talin rod into the recruitment of additional adapters (Yao et al., 2016; Rahikainen et al., 2017; Goult et al., 2018). Importantly, the role of talin in integrin activation is distinct from its mechano-transducing function (Austen et al., 2015; Rahikainen et al., 2017). In fact, the binding of a talin head only construct lacking actin binding capacity and the ability to transmit mechanical force is sufficient to induce integrin “inside-out” activation and clustering, in the absence of mechano-transmission and FA-dependent signaling (Cluzel et al., 2005; Zhang X. et al., 2008; Saltel et al., 2009; Rahikainen et al., 2017; Keeble et al., 2019; Kukkurainen et al., 2020).

### Kindlin

Proteins of the kindlin family, also known as FERMT proteins, have a structure similar to the talin head, with F0, F1, F2 and F3 domains, and a largely unstructured F1-loop (Li et al., 2017; Zhang et al., 2020) (Figure 1). In addition, within the F2 domain of kindlin is inserted a PH (pleckstrin homology) domain that recognizes membrane phosphoinositides PIP_2_ and PIP_3_ (Liu et al., 2011; Liu et al., 2012). The F3 domain of kindlin binds to the membrane-distal NITY motif and the preceding *ß*-sheet of the *β*3 integrin cytoplasmic tail (Moser et al., 2008; Harburger et al., 2009; Li et al., 2017; Bachmann et al., 2019) (Figure 1). An indirect binding of kindlin to F-actin is mediated by the ILK/Pinch/Parvin (IPP) complex (Nikolopoulos and Turner, 2000; Honda et al., 2013; Kadry et al., 2018). However, a direct interaction of kindlin with F-actin was also suggested by pull down assays (Bledzka et al., 2016). Like talin, kindlin is essential for integrin activation (Montanez et al., 2008; Moser et al., 2008; Harburger et al., 2009; Theodosiou et al., 2016; Hirbawi et al., 2017; Li et al., 2017) and cell spreading, in a mechanism proposed to be mediated by its binding to paxillin (Theodosiou et al., 2016). Mechano-transduction of kindlin within FAs appears to be linked to its ability of inducing the talin head-mediated activation and clustering of integrins (Kukkurainen et al., 2020), an essential step in the assembly of FAs (Gao et al., 2017; Li et al., 2017). In the sequence of events leading to integrin activation and clustering, it is proposed that membrane-associated kindlin assures the initial integrin-recognition event (Figure 2A), which is followed by the talin recruitment and immobilisation of the integrin-talin1-kindlin complex within FAs (Orre et al., 2019).

### Paxillin

Paxillin is a fundamental FA-associated adapter that connects structural and signaling components (Figure 2B), including tyrosine and serine/threonine kinases and GAPs/GEFs (Schaller, 2001). This hub protein coordinates integrin-downstream signaling pathways (Zaidel-Bar et al., 2007a; Green and Brown, 2019), contributing to cell spreading (Wade et al., 2002; Brimer et al., 2014; Pinon et al., 2014), migration, and proliferation (Deakin and Turner, 2008). In addition to its physiological role, paxillin sustains pathological processes in cancer progression (Lopez-Colome et al., 2017), including cell invasion (Iwasaki et al., 2002), metastasis (Ito et al., 2000) and angiogenesis (German et al., 2014).

Paxillin is composed of two modules (Figure 1): an unstructured amino-terminal half, comprising five leucine- and aspartic acid-rich motifs (with the consensus LDXLLXXL and thus named LD) forming short amphipathic *α*-helices (Bertolucci et al., 2005); and a carboxyl-terminal half composed of four LIM domains, each folded in two consecutive zinc fingers (Freyd et al., 1990; Velyvis et al., 2001). Recruitment of paxillin to FAs is mediated by the array of LIM domains, while its signaling capacity mostly relies on the N-terminal LD motif containing sequences (Brown et al., 1996; Ripamonti et al., 2021) (Figure 2B).

Although several interactions of paxillin with the elements of the β3 integrin-talin1-kindlin complex have been reported (Zacharchenko et al., 2016; Bottcher et al., 2017; Gao et al., 2017; Gough and Goult, 2018; Zhu C. et al., 2019; Atherton et al., 2020), a comprehensive understanding of paxillin engagement with this protein complex is missing. While the region of paxillin interacting with the talin F2/F3 subdomain remains unclear (Gao et al., 2017), the short helices of LD1 and LD2 can both pack against the side of the talin R8 four-helix bundle (Zacharchenko et al., 2016; Gough and Goult, 2018) (Figure 1). This mechanism of talin-binding is exploited also by other FA proteins (*e.g.,* Rho GAP, DLC1) to interact with the talin rod, suggesting that competitive interactions among different LD-motif binding proteins, such as FAK, vinculin and talin can take place within FAs (Zacharchenko et al., 2016).

Several reports have suggested that paxillin also interacts with kindlin to promote integrin activation and cell spreading (Bottcher et al., 2017; Zhu L. et al., 2019). A direct binding of the paxillin LIM3 domain to the PH domain of kindlin was proposed, based on co-immunoprecipitation experiments, deletion mutagenesis and binding assays (Theodosiou et al., 2016). In addition, interactions between the N-terminal LD motifs of paxillin and the PH and F0 domains of kindlin2, as well as between the F0 domain and the paxillin LIM3-LIM4 domains, have been identified by lysine cross-linking proteomic experiments of recombinant kindlin2-paxillin complexes (Bottcher et al., 2017). These apparently conflicting data may represent different maturation stages of FAs. It is also possible that the exceptional abundance of lysine residues within the paxillin LD motifs and the LIM4 domain revealed interactions that are only short lived or not occurring in a physiological context. The NMR structure of the kindlin F0 domain complexed with paxillin LIM4 domain (Zhu L. et al., 2019) is consistent with the recently proposed orientation of paxillin within the FA complex, and with the interaction of its positively charged LIM4 domain with the plasma membrane (Kanchanawong et al., 2010; Ripamonti et al., 2021) (Figure 1). Interestingly, the disposition of proteins within adhesions has been also addressed by means of a proximity biotinylation assay (BioID), which revealed that the paxillin N-terminus could extend for ∼25 nm into the cytoplasm, and accommodate interactions within the intermediate zone of FAs, where are situated proteins that cannot be detected by using kindlin2 as BioID probe (Dong et al., 2016). All this is consistent with iPALM studies showing that N-terminally tagged paxillin is farther away from the PM compared to the C-terminally-tagged protein (Kanchanawong et al., 2010).

The interaction of paxillin with β3 integrin is still controversial: although reported two decades ago (Pfaff and Jurdic, 2001), several biochemical experiments failed to detect the direct binding of paxillin to the cytoplasmic tail of *β*3 (Brown et al., 1996; de Curtis and Malanchini, 1997; Tanaka et al., 2010). According to the tension-dependent recruitment of paxillin to FAs and stressed actin filaments (Sawada and Sheetz, 2002; Schiller et al., 2011; Sun et al., 2020; Winkelman et al., 2020), these results may be explained by the lack of tension and proper presentation of crucial integrin residues, required for paxillin binding (Pinon et al., 2014; Ripamonti et al., 2021). Different LIM domain-mediated protein-protein interactions involve the recognition of Tyr-containing motifs by the aromatic pocket of LIM domains (Wu and Gill, 1994; Wixler et al., 2000). By analogy, it was proposed that paxillin is binding to the membrane-proximal NPLY motif of β3 integrin (Pinon et al., 2014; Soto-Ribeiro et al., 2019; Ripamonti et al., 2021). Interestingly, modification of the talin1-binding NPLY sequence led to the loss of paxillin recruitment at FAs, and to a delay in cell spreading (Wegener et al., 2007; Pinon et al., 2014; Soto-Ribeiro et al., 2019).

The intricate interplay between paxillin and FA components is exemplified by the observation that none of the *in vitro* protein-protein interactions identified so far is strictly required or sufficient for paxillin recruitment to FAs in living cells (Ripamonti et al., 2021). Conversely, a multitude of low-affinity interactions could contribute to paxillin localization at FAs and/or nascent adhesions prior to tensional force generation. For instance: the kindlin F0 binding to paxillin LIM4 domain was proposed to mediate paxillin recruitment to the plasma membrane at sites of FA assembly (Zhu L. et al., 2019); similarly the dynamic and transient binding of the paxillin LIM4 domain to the plasma membrane was shown to stabilize paxillin docking to FAs (Ripamonti et al., 2021) (Figure 1). Furthermore, a tension-independent paxillin binding to talin was disclosed by the employment of a mitochondrial targeting assay (Atherton et al., 2020), and a solid-phase binding assay (Ripamonti et al., 2021). This interaction could be functionally similar to the binding of paxillin LIM3 to the PH domain of kindlin, which was suggested to drive paxillin recruitment into nascent adhesions but not into mature FAs (Theodosiou et al., 2016). To which extent each of these interactions contributes to the stable docking of paxillin within FAs was recently addressed by measuring the off-rate of engineered paxillin molecules photoactivated within FAs in living cells (Ripamonti et al., 2021). This study confirmed the presence of a multitude of low-affinity interactions leading to paxillin FA-localization, and a complex interplay of LIM1, LIM2 and LIM4 domains to get paxillin stabilized within mature FAs.

## Paxillin as a Central Hub Mediating Mechano-Transduction at FAs

Integrin-mediated adhesions are described as mechano-sensitive because of their changes in response to mechanical stimuli (Hytonen and Wehrle-Haller, 2016; Gauthier and Roca-Cusachs, 2018). However, adhesions also fulfil the role of mechano-transducer, transmitting physical and mechanical signals from the ECM to the cytoskeleton, and converting them into cellular responses (Wehrle-Haller, 2012; Stutchbury et al., 2017). This function of FAs relies on the presence of intracellular proteins capable of sensing force-induced conformational changes, as observed for the talin and kindlin adapters (Stutchbury et al., 2017; Bachmann et al., 2019).

Several reports described the mechano-sensitivity of paxillin, although the molecular basis of its force sensing capacity is at the present not fully understood. The presence of talin and kindlin for the arrival of paxillin at nascent adhesions is necessary but not sufficient, since the development of force across the adhesion complex is also required (Cluzel et al., 2005; Hytonen and Wehrle-Haller, 2014) (Figure 2A). Along this line, it was shown that paxillin exhibits a stretch-dependent binding to the cytoskeleton (Sawada and Sheetz, 2002), as well as a remarkable ability of its LIM domains to detect mechanically strained stress fibers (Smith et al., 2013). Furthermore, several LIM domain-containing proteins that cluster at FAs (Kadrmas and Beckerle, 2004) are recruited in a myosin II-dependent fashion, suggesting that LIM domains could function as tension sensors of a strained F-actin network (Schiller et al., 2011; Sun et al., 2020; Winkelman et al., 2020).

The tension-dependent binding of paxillin to adhesions, combined to its hub function, suggests a major role of this protein in integrating signals from the integrin complex and in activating molecular pathways shaping cell behaviour (Green and Brown, 2019) (Figure 2B). The versatility of paxillin in the selection of binding partners is supported by the nature of its LD domains (Alam et al., 2020) that generally establish poorly selective, transient interactions, which require multiple layers of regulation (Alam et al., 2014). Due to the low binding affinity of single LD motifs, multiple simultaneous interactions are required to achieve stable complexes and elicit cellular responses (Alam et al., 2014). For example, opposite faces of the four-helix bundle in the FAT (Focal Adhesion Targeting) domain of FAK and in vinculin tail associate to paxillin LD2 and LD4 (Hoellerer et al., 2003).

Owing to its extraordinary connection with a plethora of adhesome components, paxillin is regarded as a unique protein capable of integrating the diverse functions of FAs (Green and Brown, 2019; Chastney et al., 2020). In other words, paxillin fulfils the crucial linker function connecting the core actin, the cell cortex, the signaling, and the regulatory modules constituted by subsets of FA proteins (Figure 2B) (Green and Brown, 2019; Chastney et al., 2020). For a complete understanding of paxillin interactions and functions, precise analyses considering FA protein isoforms and their post-translational modifications should be considered as well. The analysis of these aspects goes beyond the goal of this review, yet we can provide as an example the cell adhesion-triggered paxillin phosphorylation at Tyr^31^ and Tyr^118^ (Burridge et al., 1992) which modulates its binding to *β*3 integrin adhesions (Ripamonti et al., 2021), possibly by increasing paxillin affinity for FAK and vinculin (Zaidel-Bar et al., 2007b; Choi et al., 2011; Case et al., 2015). Noteworthy, the described paxillin-dependent nanoscale (re-)localization of vinculin within the FA architecture (Case et al., 2015) suggests that paxillin functions as a FA organizer beside its linker function (Figure 2B) (Green and Brown, 2019).

How tension and the assembly of the described *β*3 integrin-talin1-kindlin-paxillin complex at FAs is dynamically regulated during cell motility remains an open question. Adhesion remodelling directly and positively correlates with the ability of cells to migrate (Deakin and Turner, 2011), which physically relies on adhesion formation at the leading edge and adhesion disassembly at the cell rear (Webb et al., 2004; Cluzel et al., 2005). The latter was proposed to be under the control of Src-mediated phosphorylation of paxillin Tyr^31/118^ (Cortesio et al., 2011). Accordingly, Tyr-to-Phe mutations of these residues hampered adhesion turnover (Webb et al., 2004; Zaidel-Bar et al., 2007b) and inhibited tumor cell invasion (Mekhdjian et al., 2017). Consistent with these findings, sustained paxillin binding to FAs, phosphorylation of Tyr^31/118^, and FAK signaling can result in FA disassembly and turnover of its components (Webb et al., 2004). On the other hand, loss of paxillin phosphorylation was proposed to be responsible of hindering FA disassembly and support FA maturation toward fibrillar adhesions and their translocation to the cell center (Zaidel-Bar et al., 2007b; Bachmann et al., 2019).

Paxillin may be involved in the regulation of tension at the cell edge during migration on ECM ligands. In this direction, the complex between the ArfGAP and scaffold protein GIT1 (G-protein-coupled receptor-kinase interacting protein-1) and the guanine nucleotide exchange factor for Rac1 βPix has been implicated in the regulation of FAs and cell migration (Turner et al., 1999; Premont et al., 2000). GIT1 is recruited to FAs by direct binding of its FA-targeting domain to paxillin LD2 and LD4 motifs (Schmalzigaug et al., 2007; Zhang Z. M. et al., 2008; Wehrle-Haller and Bastmeyer, 2014). Recently, evidence has been provided for the formation of protein condensates of the GIT1/βPix complex driven by liquid-liquid phase separation (Zhu et al., 2020), a process involved in the organization and compartmentalization of several events occurring in the cytoplasm and the nucleus of eukaryotic cells (Banani et al., 2017; de Curtis, 2021). The results from the study of the Zhang’s group indicate that the formation of GIT1/βPix condensates and their targeting at FAs by paxillin are required to regulate cell migration (Zhu et al., 2020). Paxillin is shown to promote the formation of GIT1/βPix condensates, and one intriguing hypothesis is that paxillin-mediated formation and recruitment of GIT1/βPix condensates at FAs may modulate F-actin polymerization and tension within adhesions to modulate FAs turnover (Figure 2B). Also, dominant-active Rac1-transfected cells presented slower integrin turnover than control cells, indicating that βPix activity may locally stabilize the turnover of integrins, and arrest retrograde sliding adhesions (Ballestrem et al., 2001). These mechanisms could explain the paxillin-mediated rescue of unstable and rapidly sliding adhesions, indicating a role of paxillin in the control of the F-actin feedback loop (Ripamonti et al., 2021).

## Conclusion and Perspectives

The gathering of structural and positional data led to the proposal of the slanted fence model of FAs, in which connections among neighbouring integrin-talin1-kindlin-paxillin units stabilize the complex (Sun et al., 2016; Bachmann et al., 2019; Ripamonti et al., 2021) (Figure 1). The proposed layered organization of FAs potentially bears the secret how integrin receptors support mechanical load and create intracellular signaling in response to changes in tensional forces (Kanchanawong et al., 2010; Bachmann et al., 2019). The detailed characterization of this key biological mechanism, tightly related to paxillin recruitment and binding to FAs (Figure 2) (Cluzel et al., 2005; Hytonen and Wehrle-Haller, 2014, 2016; Ripamonti et al., 2021), will help in the development of efficient integrin-targeting anti-cancer therapies, so far challenged by the complexity of the integrin system which has caused unexpected side effects (Su et al., 2020; Li et al., 2021).

## References

[B1] Alam T. Alazmi M. Naser R. Huser F. Momin A. A. Astro V. (2020). Proteome-level Assessment of Origin, Prevalence and Function of Leucine-Aspartic Acid (LD) Motifs. Bioinformatics 36, 1121–1128. 10.1093/bioinformatics/btz703 31584626 PMC7703752

[B2] Alam T. Alazmi M. Gao X. Arold S. T. (2014). How to Find a Leucine in a Haystack? Structure, Ligand Recognition and Regulation of Leucine-Aspartic Acid (LD) Motifs. Biochem. J. 460, 317–329. 10.1042/bj20140298 24870021

[B3] Anthis N. J. Wegener K. L. Critchley D. R. Campbell I. D. (2010). Structural Diversity in Integrin/talin Interactions. Structure 18, 1654–1666. 10.1016/j.str.2010.09.018 21134644 PMC3157975

[B4] Anthis N. J. Wegener K. L. Ye F. Kim C. Goult B. T. Lowe E. D. (2009). The Structure of an Integrin/talin Complex Reveals the Basis of Inside-Out Signal Transduction. EMBO J. 28, 3623–3632. 10.1038/emboj.2009.287 19798053 PMC2782098

[B5] Atherton P. Lausecker F. Carisey A. Gilmore A. Critchley D. Barsukov I. (2020). Relief of Talin Autoinhibition Triggers a Force-independent Association with Vinculin. J. Cel Biol 219, e201903134. 10.1083/jcb.201903134 PMC7039207 31816055

[B6] Attieh Y. Clark A. G. Grass C. Richon S. Pocard M. Mariani P. (2017). Cancer-associated Fibroblasts lead Tumor Invasion through Integrin-β3-dependent Fibronectin Assembly. J. Cel Biol 216, 3509–3520. 10.1083/jcb.201702033 PMC5674886 28931556

[B7] Austen K. Ringer P. Mehlich A. Chrostek-Grashoff A. Kluger C. Klingner C. (2015). Extracellular Rigidity Sensing by Talin Isoform-specific Mechanical Linkages. Nat. Cel Biol 17, 1597–1606. 10.1038/ncb3268 PMC4662888 26523364

[B8] Bachmann M. Schäfer M. Mykuliak V. V. Ripamonti M. Heiser L. Weissenbruch K. (2020). Induction of Ligand Promiscuity of αVβ3 Integrin by Mechanical Force. J. Cel Sci 133, jcs242404. 10.1242/jcs.242404 32193334

[B9] Bachmann M. Kukkurainen S. Hytönen V. P. Wehrle-Haller B. (2019). Cell Adhesion by Integrins. Physiol. Rev. 99, 1655–1699. 10.1152/physrev.00036.2018 31313981

[B10] Ballestrem C. Hinz B. Imhof B. A. Wehrle-Haller B. (2001). Marching at the Front and Dragging behind. J. Cel Biol 155, 1319–1332. 10.1083/jcb.200107107 PMC2199321 11756480

[B11] Banani S. F. Lee H. O. Hyman A. A. Rosen M. K. (2017). Biomolecular Condensates: Organizers of Cellular Biochemistry. Nat. Rev. Mol. Cel Biol 18, 285–298. 10.1038/nrm.2017.7 PMC7434221 28225081

[B12] Bertolucci C. M. Guibao C. D. Zheng J. (2005). Structural Features of the Focal Adhesion Kinase-Paxillin Complex Give Insight into the Dynamics of Focal Adhesion Assembly. Protein Sci. 14, 644–652. 10.1110/ps.041107205 15689512 PMC2279287

[B13] Bledzka K. Bialkowska K. Sossey-Alaoui K. Vaynberg J. Pluskota E. Qin J. (2016). Kindlin-2 Directly Binds Actin and Regulates Integrin Outside-In Signaling. J. Cel Biol 213, 97–108. 10.1083/jcb.201501006 PMC4828686 27044892

[B14] Böttcher R. T. Veelders M. Rombaut P. Faix J. Theodosiou M. Stradal T. E. (2017). Kindlin-2 Recruits Paxillin and Arp2/3 to Promote Membrane Protrusions during Initial Cell Spreading. J. Cel Biol 216, 3785–3798. 10.1083/jcb.201701176 PMC5674885 28912124

[B15] Brimer N. Wade R. Vande Pol S. (2014). Interactions between E6, FAK, and GIT1 at Paxillin LD4 Are Necessary for Transformation by Bovine Papillomavirus 1 E6. J. Virol. 88, 9927–9933. 10.1128/jvi.00552-14 24942580 PMC4136348

[B16] Brooks P. C. Strömblad S. Sanders L. C. Von Schalscha T. L. Aimes R. T. Stetler-Stevenson W. G. (1996). Localization of Matrix Metalloproteinase MMP-2 to the Surface of Invasive Cells by Interaction with Integrin αvβ3. Cell 85, 683–693. 10.1016/s0092-8674(00)81235-0 8646777

[B17] Brown M. C. Perrotta J. A. Turner C. E. (1996). Identification of LIM3 as the Principal Determinant of Paxillin Focal Adhesion Localization and Characterization of a Novel Motif on Paxillin Directing Vinculin and Focal Adhesion Kinase Binding. J. Cel Biol 135, 1109–1123. 10.1083/jcb.135.4.1109 PMC2133378 8922390

[B18] Burridge K. Turner C. E. Romer L. H. (1992). Tyrosine Phosphorylation of Paxillin and pp125FAK Accompanies Cell Adhesion to Extracellular Matrix: a Role in Cytoskeletal Assembly. J. Cel Biol 119, 893–903. 10.1083/jcb.119.4.893 PMC2289706 1385444

[B19] Calderwood D. A. Campbell I. D. Critchley D. R. (2013). Talins and Kindlins: Partners in Integrin-Mediated Adhesion. Nat. Rev. Mol. Cel Biol 14, 503–517. 10.1038/nrm3624 PMC4116690 23860236

[B20] Case L. B. Baird M. A. Shtengel G. Campbell S. L. Hess H. F. Davidson M. W. (2015). Molecular Mechanism of Vinculin Activation and Nanoscale Spatial Organization in Focal Adhesions. Nat. Cel Biol 17, 880–892. 10.1038/ncb3180 PMC4490039 26053221

[B21] Chastney M. R. Lawless C. Humphries J. D. Warwood S. Jones M. C. Knight D. (2020). Topological Features of Integrin Adhesion Complexes Revealed by Multiplexed Proximity Biotinylation. J. Cel Biol 219, e202003038. 10.1083/jcb.202003038 PMC7401799 32585685

[B22] Choi C. K. Zareno J. Digman M. A. Gratton E. Horwitz A. R. (2011). Cross-correlated Fluctuation Analysis Reveals Phosphorylation-Regulated Paxillin-FAK Complexes in Nascent Adhesions. Biophysical J. 100, 583–592. 10.1016/j.bpj.2010.12.3719 PMC3030238 21281572

[B23] Cluzel C. Saltel F. Lussi J. Paulhe F. Imhof B. A. Wehrle-Haller B. (2005). The Mechanisms and Dynamics of αvβ3 Integrin Clustering in Living Cells. J. Cel Biol 171, 383–392. 10.1083/jcb.200503017 PMC2171205 16247034

[B24] Cortesio C. L. Boateng L. R. Piazza T. M. Bennin D. A. Huttenlocher A. (2011). Calpain-mediated Proteolysis of Paxillin Negatively Regulates Focal Adhesion Dynamics and Cell Migration. J. Biol. Chem. 286, 9998–10006. 10.1074/jbc.m110.187294 21270128 PMC3060554

[B25] de Curtis I. (2021). Biomolecular Condensates at the Front: Cell Migration Meets Phase Separation. Trends Cel Biol. 31, 145–148. 10.1016/j.tcb.2020.12.002 33397597

[B26] de Curtis I. Malanchini B. (1997). Integrin-mediated Tyrosine Phosphorylation and Redistribution of Paxillin during Neuronal Adhesion. Exp. Cel Res. 230, 233–243. 10.1006/excr.1996.3423 9024782

[B27] Deakin N. O. Turner C. E. (2011). Distinct Roles for Paxillin and Hic-5 in Regulating Breast Cancer Cell Morphology, Invasion, and Metastasis. MBoC 22, 327–341. 10.1091/mbc.e10-09-0790 21148292 PMC3031464

[B28] Deakin N. O. Turner C. E. (2008). Paxillin Comes of Age. J. Cel Sci 121, 2435–2444. 10.1242/jcs.018044 PMC2522309 18650496

[B29] Del Rio A. Perez-Jimenez R. Liu R. Roca-Cusachs P. Fernandez J. M. Sheetz M. P. (2009). Stretching Single Talin Rod Molecules Activates Vinculin Binding. Science 323, 638–641. 10.1126/science.1162912 19179532 PMC9339221

[B30] Dong J. M. Tay F. P. Swa H. L. Gunaratne J. Leung T. Burke B. (2016). Proximity Biotinylation Provides Insight into the Molecular Composition of Focal Adhesions at the Nanometer Scale. Sci. Signal. 9, rs4. 10.1126/scisignal.aaf3572 27303058

[B31] Freyd G. Kim S. K. Horvitz H. R. (1990). Novel Cysteine-Rich Motif and Homeodomain in the Product of the *Caenorhabditis elegans* Cell Lineage Gene Lin-II. Nature 344, 876–879. 10.1038/344876a0 1970421

[B32] Gao J. Huang M. Lai J. Mao K. Sun P. Cao Z. (2017). Kindlin Supports Platelet Integrin αIIbβ3 Activation by Interacting with Paxillin. J. Cel Sci 130, 3764–3775. 10.1242/jcs.205641 PMC6040092 28954813

[B33] Gauthier N. C. Roca-Cusachs P. (2018). Mechanosensing at Integrin-Mediated Cell-Matrix Adhesions: from Molecular to Integrated Mechanisms. Curr. Opin. Cel Biol. 50, 20–26. 10.1016/j.ceb.2017.12.014 29438903

[B34] Geiger B Yamada KM (2011). Molecular architecture and function of matrix adhesions. Cold Spring Harb Perspect Biol 3, a005033. 10.1101/cshperspect.a005033 21441590 PMC3101841

[B35] German A. E. Mammoto T. Jiang E. Ingber D. E. Mammoto A. (2014). Paxillin controls endothelial cell migration and tumor angiogenesis by altering neuropilin 2 expression. J Cell Sci 127, 1672–1683. 10.1242/jcs.132316 24522185 PMC3986673

[B36] Goksoy E. Ma Y.-Q. Wang X. Kong X. Perera D. Plow E. F. Qin J. (2008). Structural basis for the autoinhibition of talin in regulating integrin activation. Molecular Cell 31, 124–133. 10.1016/j.molcel.2008.06.011 18614051 PMC2522368

[B37] Gough R. E. Goult B. T. (2018). The Tale of Two Talins - Two Isoforms to fine-tune Integrin Signalling. FEBS Lett. 592, 2108–2125. 10.1002/1873-3468.13081 29723415 PMC6032930

[B38] Goult B. T. Bouaouina M. Elliott P. R. Bate N. Patel B. Gingras A. R. (2010). Structure of a Double Ubiquitin-like Domain in the Talin Head: a Role in Integrin Activation. EMBO J. 29, 1069–1080. 10.1038/emboj.2010.4 20150896 PMC2845276

[B39] Goult B. T. Yan J. Schwartz M. A. (2018). Talin as a Mechanosensitive Signaling Hub. J. Cel Biol 217, 3776–3784. 10.1083/jcb.201808061 PMC6219721 30254032

[B40] Green H. J. Brown N. H. (2019). Integrin Intracellular Machinery in Action. Exp. Cel Res. 378, 226–231. 10.1016/j.yexcr.2019.03.011 30853446

[B41] Harburger D. S. Bouaouina M. Calderwood D. A. (2009). Kindlin-1 and -2 Directly Bind the C-Terminal Region of β Integrin Cytoplasmic Tails and Exert Integrin-specific Activation Effects. J. Biol. Chem. 284, 11485–11497. 10.1074/jbc.m809233200 19240021 PMC2670154

[B42] Havaki S. Kouloukoussa M. Amawi K. Drosos Y. Arvanitis L. D. Goutas N. (2007). Altered Expression Pattern of Integrin Alphavbeta3 Correlates with Actin Cytoskeleton in Primary Cultures of Human Breast Cancer. Cancer Cel Int 7, 16. 10.1186/1475-2867-7-16 PMC2116995 17910753

[B43] Hirbawi J. Bialkowska K. Bledzka K. M. Liu J. Fukuda K. Qin J. (2017). The Extreme C-Terminal Region of Kindlin-2 Is Critical to its Regulation of Integrin Activation. J. Biol. Chem. 292, 14258–14269. 10.1074/jbc.m117.776195 28652408 PMC5572925

[B44] Hoellerer M. K. Noble M. E. M. Labesse G. Campbell I. D. Werner J. M. Arold S. T. (2003). Molecular Recognition of Paxillin LD Motifs by the Focal Adhesion Targeting Domain. Structure 11, 1207–1217. 10.1016/j.str.2003.08.010 14527389

[B45] Honda S. Shirotani-Ikejima H. Tadokoro S. Tomiyama Y. Miyata T. (2013). The Integrin-Linked Kinase-PINCH-Parvin Complex Supports Integrin αIIbβ3 Activation. PLoS One 8, e85498. 10.1371/journal.pone.0085498 24376884 PMC3871693

[B46] Horton M. A. (1997). The αvβ3 Integrin "vitronectin Receptor". Int. J. Biochem. Cel Biol. 29, 721–725. 10.1016/s1357-2725(96)00155-0 9251239

[B47] Huang J. Li X. Shi X. Zhu M. Wang J. Huang S. (2019). Platelet Integrin αIIbβ3: Signal Transduction, Regulation, and its Therapeutic Targeting. J. Hematol. Oncol. 12, 26. 10.1186/s13045-019-0709-6 30845955 PMC6407232

[B48] Humphries J. D. Byron A. Humphries M. J. (2006). Integrin Ligands at a Glance. J. Cel Sci 119, 3901–3903. 10.1242/jcs.03098 PMC3380273 16988024

[B49] Humphries J. D. Wang P. Streuli C. Geiger B. Humphries M. J. Ballestrem C. (2007). Vinculin Controls Focal Adhesion Formation by Direct Interactions with Talin and Actin. J. Cel Biol 179, 1043–1057. 10.1083/jcb.200703036 PMC2099183 18056416

[B50] Huveneers S. Van Den Bout I. Sonneveld P. Sancho A. Sonnenberg A. Danen E. H. J. (2007). Integrin αvβ3 Controls Activity and Oncogenic Potential of Primed C-Src. Cancer Res. 67, 2693–2700. 10.1158/0008-5472.can-06-3654 17363590

[B51] Hynes R. O. (2002). Integrins. Cell 110, 673–687. 10.1016/s0092-8674(02)00971-6 12297042

[B52] Hytönen V. P. Vogel V. (2008). How Force Might Activate Talin's Vinculin Binding Sites: SMD Reveals a Structural Mechanism. Plos Comput. Biol. 4, e24. 10.1371/journal.pcbi.0040024 18282082 PMC2242828

[B53] Hytönen V. P. Wehrle-Haller B. (2016). Mechanosensing in Cell-Matrix Adhesions - Converting Tension into Chemical Signals. Exp. Cel Res. 343, 35–41. 10.1016/j.yexcr.2015.10.027 26518118

[B54] Hytönen V. P. Wehrle-Haller B. (2014). Protein Conformation as a Regulator of Cell-Matrix Adhesion. Phys. Chem. Chem. Phys. 16, 6342–6357. 10.1039/c3cp54884h 24469063

[B55] Ito A. Kataoka T. R. Watanabe M. Nishiyama K. Mazaki Y. Sabe H. (2000). A Truncated Isoform of the PP2A B56 Subunit Promotes Cell Motility through Paxillin Phosphorylation. EMBO J. 19, 562–571. 10.1093/emboj/19.4.562 10675325 PMC305594

[B56] Iwasaki T. Nakata A. Mukai M. Shinkai K. Yano H. Sabe H. (2002). Involvement of Phosphorylation of Tyr-31 and Tyr-118 of Paxillin in MM1 Cancer Cell Migration. Int. J. Cancer 97, 330–335. 10.1002/ijc.1609 11774284

[B57] Kadrmas J. L. Beckerle M. C. (2004). The LIM Domain: from the Cytoskeleton to the Nucleus. Nat. Rev. Mol. Cel Biol 5, 920–931. 10.1038/nrm1499 15520811

[B58] Kadry Y. A. Huet-Calderwood C. Simon B. Calderwood D. A. (2018). Kindlin-2 Interacts with a Highly Conserved Surface of ILK to Regulate Focal Adhesion Localization and Cell Spreading. J. Cel Sci 131, jcs221184. 10.1242/jcs.221184 PMC6215391 30254023

[B59] Kanchanawong P. Shtengel G. Pasapera A. M. Ramko E. B. Davidson M. W. Hess H. F. (2010). Nanoscale Architecture of Integrin-Based Cell Adhesions. Nature 468, 580–584. 10.1038/nature09621 21107430 PMC3046339

[B60] Keeble A. H. Turkki P. Stokes S. Khairil Anuar I. N. A. Rahikainen R. Hytonen V. P. (2019). Approaching Infinite Affinity through Engineering of Peptide-Protein Interaction. Proc Natl Acad Sci U S A. 10.1073/pnas.1909653116 PMC6936558 31822621

[B61] Kukkurainen S. Azizi L. Zhang P. Jacquier M. C. Baikoghli M. Von Essen M. (2020). The F1 Loop of the Talin Head Domain Acts as a Gatekeeper in Integrin Activation and Clustering. J. Cel Sci 133, jcs239202. 10.1242/jcs.239202 PMC10679385 33046605

[B62] Lagarrigue F. Paul D. S. Gingras A. R. Valadez A. J. Sun H. Lin J. (2020). Talin-1 Is the Principal Platelet Rap1 Effector of Integrin Activation. Blood 136, 1180–1190. 10.1182/blood.2020005348 32518959 PMC7472713

[B63] Legerstee K. Houtsmuller A. B. (2021). A Layered View on Focal Adhesions. Biology (Basel) 10, 1189. 10.3390/biology10111189 34827182 PMC8614905

[B64] Li H. Deng Y. Sun K. Yang H. Liu J. Wang M. (2017). Structural Basis of Kindlin-Mediated Integrin Recognition and Activation. Proc. Natl. Acad. Sci. U.S.A. 114, 9349–9354. 10.1073/pnas.1703064114 28739949 PMC5584418

[B65] Li M. Wang Y. Li M. Wu X. Setrerrahmane S. Xu H. (2021). Integrins as Attractive Targets for Cancer Therapeutics. Acta Pharmaceutica Sinica B 11, 2726–2737. 10.1016/j.apsb.2021.01.004 34589393 PMC8463276

[B66] Liu J. Fukuda K. Xu Z. Ma Y.-Q. Hirbawi J. Mao X. (2011). Structural Basis of Phosphoinositide Binding to Kindlin-2 Protein Pleckstrin Homology Domain in Regulating Integrin Activation. J. Biol. Chem. 286, 43334–43342. 10.1074/jbc.m111.295352 22030399 PMC3234820

[B67] Liu Y. Zhu Y. Ye S. Zhang R. (2012). Crystal Structure of Kindlin-2 PH Domain Reveals a Conformational Transition for its Membrane Anchoring and Regulation of Integrin Activation. Protein Cell 3, 434–440. 10.1007/s13238-012-2046-1 22653426 PMC4875484

[B68] López-Colomé A. M. Lee-Rivera I. Benavides-Hidalgo R. López E. (2017). Paxillin: a Crossroad in Pathological Cell Migration. J. Hematol. Oncol. 10, 50. 10.1186/s13045-017-0418-y 28214467 PMC5316197

[B69] Mahabeleshwar G. H. Feng W. Reddy K. Plow E. F. Byzova T. V. (2007). Mechanisms of Integrin-Vascular Endothelial Growth Factor Receptor Cross-Activation in Angiogenesis. Circ. Res. 101, 570–580. 10.1161/circresaha.107.155655 17641225 PMC2723825

[B70] Mekhdjian A. H. Kai F. Rubashkin M. G. Prahl L. S. Przybyla L. M. Mcgregor A. L. (2017). Integrin-mediated Traction Force Enhances Paxillin Molecular Associations and Adhesion Dynamics that Increase the Invasiveness of Tumor Cells into a Three-Dimensional Extracellular Matrix. MBoC 28, 1467–1488. 10.1091/mbc.e16-09-0654 28381423 PMC5449147

[B71] Montanez E. Ussar S. Schifferer M. Bösl M. Zent R. Moser M. (2008). Kindlin-2 Controls Bidirectional Signaling of Integrins. Genes Dev. 22, 1325–1330. 10.1101/gad.469408 18483218 PMC2377186

[B72] Moser M. Nieswandt B. Ussar S. Pozgajova M. Fässler R. (2008). Kindlin-3 Is Essential for Integrin Activation and Platelet Aggregation. Nat. Med. 14, 325–330. 10.1038/nm1722 18278053

[B73] Nikolopoulos S. N. Turner C. E. (2000). Actopaxin, a New Focal Adhesion Protein that Binds Paxillin LD Motifs and Actin and Regulates Cell Adhesion. J. Cel Biol 151, 1435–1448. 10.1083/jcb.151.7.1435 PMC2150668 11134073

[B74] Orré T. Joly A. Karatas Z. Kastberger B. Cabriel C. Böttcher R. T. (2021). Molecular Motion and Tridimensional Nanoscale Localization of Kindlin Control Integrin Activation in Focal Adhesions. Nat. Commun. 12, 3104. 10.1038/s41467-021-23372-w 34035280 PMC8149821

[B75] Orré T. Rossier O. Giannone G. (2019). The Inner Life of Integrin Adhesion Sites: From Single Molecules to Functional Macromolecular Complexes. Exp. Cel Res. 379, 235–244. 10.1016/j.yexcr.2019.03.036 30943383

[B76] Pfaff M. Jurdic P. (2001). Podosomes in Osteoclast-like Cells. J. Cel Sci 114, 2775–2786. 10.1242/jcs.114.15.2775 11683411

[B77] Pinon P. Pärssinen J. Vazquez P. Bachmann M. Rahikainen R. Jacquier M.-C. (2014). Talin-bound NPLY Motif Recruits Integrin-Signaling Adapters to Regulate Cell Spreading and Mechanosensing. J. Cel Biol 205, 265–281. 10.1083/jcb.201308136 PMC4003243 24778313

[B78] Premont R. T. Claing A. Vitale N. Perry S. J. Lefkowitz R. J. (2000). The GIT Family of ADP-Ribosylation Factor GTPase-Activating Proteins. J. Biol. Chem. 275, 22373–22380. 10.1074/jbc.275.29.22373 10896954

[B79] Pytela R. Pierschbacher M. D. Ruoslahti E. (1985). A 125/115-kDa Cell Surface Receptor Specific for Vitronectin Interacts with the Arginine-Glycine-Aspartic Acid Adhesion Sequence Derived from Fibronectin. Proc. Natl. Acad. Sci. U.S.A. 82, 5766–5770. 10.1073/pnas.82.17.5766 2412224 PMC390633

[B80] Rahikainen R. Von Essen M. Schaefer M. Qi L. Azizi L. Kelly C. (2017). Mechanical Stability of Talin Rod Controls Cell Migration and Substrate Sensing. Sci. Rep. 7, 3571. 10.1038/s41598-017-03335-2 28620171 PMC5472591

[B81] Ripamonti M. Liaudet N. Azizi L. Bouvard D. Hytönen V. P. Wehrle-Haller B. (2021). Structural and Functional Analysis of LIM Domain-dependent Recruitment of Paxillin to αvβ3 Integrin-Positive Focal Adhesions. Commun. Biol. 4, 380. 10.1038/s42003-021-01886-9 33782527 PMC8007706

[B82] Saalbach A. Wetzel A. Haustein U.-F. Sticherling M. Simon J. C. Anderegg U. (2005). Interaction of Human Thy-1 (CD 90) with the Integrin αvβ3 (CD51/CD61): an Important Mechanism Mediating Melanoma Cell Adhesion to Activated Endothelium. Oncogene 24, 4710–4720. 10.1038/sj.onc.1208559 15897908

[B83] Saltel F. Mortier E. Hytönen V. P. Jacquier M.-C. Zimmermann P. Vogel V. (2009). New PI(4,5)P2- and Membrane Proximal Integrin-Binding Motifs in the Talin Head Control β3-integrin Clustering. J. Cel Biol 187, 715–731. 10.1083/jcb.200908134 PMC2806581 19948488

[B84] Sawada Y. Sheetz M. P. (2002). Force Transduction by Triton Cytoskeletons. J. Cel Biol 156, 609–615. 10.1083/jcb.200110068 PMC2174068 11839769

[B85] Schaller M. D. (2001). Paxillin: a Focal Adhesion-Associated Adaptor Protein. Oncogene 20, 6459–6472. 10.1038/sj.onc.1204786 11607845

[B86] Schiller H. B. Friedel C. C. Boulegue C. Fässler R. (2011). Quantitative Proteomics of the Integrin Adhesome Show a Myosin II‐dependent Recruitment of LIM Domain Proteins. EMBO Rep. 12, 259–266. 10.1038/embor.2011.5 21311561 PMC3059911

[B87] Schmalzigaug R. Garron M. Roseman J. Xing Y. Davidson C. Arold S. (2007). GIT1 Utilizes a Focal Adhesion Targeting-Homology Domain to Bind Paxillin. Cell Signal. 19, 1733–1744. 10.1016/j.cellsig.2007.03.010 17467235 PMC2025689

[B88] Smith M. A. Blankman E. Deakin N. O. Hoffman L. M. Jensen C. C. Turner C. E. (2013). LIM Domains Target Actin Regulators Paxillin and Zyxin to Sites of Stress Fiber Strain. PLoS One 8, e69378. 10.1371/journal.pone.0069378 23990882 PMC3749209

[B89] Soto-Ribeiro M. Kastberger B. Bachmann M. Azizi L. Fouad K. Jacquier M. C. (2019). β1D Integrin Splice Variant Stabilizes Integrin Dynamics and Reduces Integrin Signaling by Limiting Paxillin Recruitment. J. Cel Sci 132, jcs224493. 10.1242/jcs.224493 30890648

[B90] Stutchbury B. Atherton P. Tsang R. Wang D. Y. Ballestrem C. (2017). Distinct Focal Adhesion Protein Modules Control Different Aspects of Mechanotransduction. J. Cel Sci 130, 1612–1624. 10.1242/jcs.195362 PMC5450230 28302906

[B91] Su C.-y. Li J.-q. Zhang L.-l. Wang H. Wang F.-h. Tao Y.-w. (2020). The Biological Functions and Clinical Applications of Integrins in Cancers. Front. Pharmacol. 11, 579068. 10.3389/fphar.2020.579068 33041823 PMC7522798

[B92] Sun X. Phua D. Y. Z. Axiotakis L. Jr. Smith M. A. Blankman E. Gong R. (2020). Mechanosensing through Direct Binding of Tensed F-Actin by LIM Domains. Developmental Cel 55, 468–482. 10.1016/j.devcel.2020.09.022 PMC7686152 33058779

[B93] Sun Z. Guo S. S. Fässler R. (2016). Integrin-mediated Mechanotransduction. J. Cel Biol 215, 445–456. 10.1083/jcb.201609037 PMC5119943 27872252

[B94] Tanaka T. Moriwaki K. Murata S. Miyasaka M. (2010). LIM Domain-Containing Adaptor, Leupaxin, Localizes in Focal Adhesion and Suppresses the Integrin-Induced Tyrosine Phosphorylation of Paxillin. Cancer Sci. 101, 363–368. 10.1111/j.1349-7006.2009.01398.x 19917054 PMC11158308

[B95] Theodosiou M. Widmaier M. Böttcher R. T. Rognoni E. Veelders M. Bharadwaj M. (2016). Kindlin-2 Cooperates with Talin to Activate Integrins and Induces Cell Spreading by Directly Binding Paxillin. Elife 5, e10130. 10.7554/eLife.10130 26821125 PMC4749545

[B96] Thievessen I. Thompson P. M. Berlemont S. Plevock K. M. Plotnikov S. V. Zemljic-Harpf A. (2013). Vinculin-actin Interaction Couples Actin Retrograde Flow to Focal Adhesions, but Is Dispensable for Focal Adhesion Growth. J. Cel Biol 202, 163–177. 10.1083/jcb.201303129 PMC3704983 23836933

[B97] Turner C. E. Brown M. C. Perrotta J. A. Riedy M. C. Nikolopoulos S. N. Mcdonald A. R. (1999). Paxillin LD4 Motif Binds PAK and PIX through a Novel 95-kD Ankyrin Repeat, ARF-GAP Protein: A Role in Cytoskeletal Remodeling. J. Cel Biol 145, 851–863. 10.1083/jcb.145.4.851 PMC2133183 10330411

[B98] Velyvis A. Yang Y. Wu C. Qin J. (2001). Solution Structure of the Focal Adhesion Adaptor PINCH LIM1 Domain and Characterization of its Interaction with the Integrin-Linked Kinase Ankyrin Repeat Domain. J. Biol. Chem. 276, 4932–4939. 10.1074/jbc.m007632200 11078733

[B99] Vogel V. (2006). Mechanotransduction Involving Multimodular Proteins: Converting Force into Biochemical Signals. Annu. Rev. Biophys. Biomol. Struct. 35, 459–488. 10.1146/annurev.biophys.35.040405.102013 16689645

[B100] Vonlaufen A. Wiedle G. Borisch B. Birrer S. Luder P. Imhof B. A. (2001). Integrin αvβ3 Expression in Colon Carcinoma Correlates with Survival. Mod. Pathol. 14, 1126–1132. 10.1038/modpathol.3880447 11706074

[B101] Wade R. Bohl J. Vande Pol S. (2002). Paxillin Null Embryonic Stem Cells Are Impaired in Cell Spreading and Tyrosine Phosphorylation of Focal Adhesion Kinase. Oncogene 21, 96–107. 10.1038/sj.onc.1205013 11791180

[B102] Webb D. J. Donais K. Whitmore L. A. Thomas S. M. Turner C. E. Parsons J. T. (2004). FAK-src Signalling through Paxillin, ERK and MLCK Regulates Adhesion Disassembly. Nat. Cel Biol 6, 154–161. 10.1038/ncb1094 14743221

[B103] Wegener K. L. Basran J. Bagshaw C. R. Campbell I. D. Roberts G. C. K. Critchley D. R. (2008). Structural Basis for the Interaction between the Cytoplasmic Domain of the Hyaluronate Receptor Layilin and the Talin F3 Subdomain. J. Mol. Biol. 382, 112–126. 10.1016/j.jmb.2008.06.087 18638481

[B104] Wegener K. L. Partridge A. W. Han J. Pickford A. R. Liddington R. C. Ginsberg M. H. (2007). Structural Basis of Integrin Activation by Talin. Cell 128, 171–182. 10.1016/j.cell.2006.10.048 17218263

[B105] Wehrle-Haller B. Bastmeyer M. (2014). Intracellular Signaling and Perception of Neuronal Scaffold through Integrins and Their Adapter Proteins. Prog. Brain Res. 214, 443–460. 10.1016/b978-0-444-63486-3.00018-9 25410368

[B106] Wehrle-Haller B. (2012). Structure and Function of Focal Adhesions. Curr. Opin. Cel Biol. 24, 116–124. 10.1016/j.ceb.2011.11.001 22138388

[B107] Winkelman J. D. Anderson C. A. Suarez C. Kovar D. R. Gardel M. L. (2020). Evolutionarily Diverse LIM Domain-Containing Proteins Bind Stressed Actin Filaments through a Conserved Mechanism. Proc. Natl. Acad. Sci. U.S.A. 117, 25532–25542. 10.1073/pnas.2004656117 32989126 PMC7568268

[B108] Winograd-Katz S. E. Fässler R. Geiger B. Legate K. R. (2014). The Integrin Adhesome: from Genes and Proteins to Human Disease. Nat. Rev. Mol. Cel Biol 15, 273–288. 10.1038/nrm3769 24651544

[B109] Wixler V. Geerts D. Laplantine E. Westhoff D. Smyth N. Aumailley M. (2000). The LIM-Only Protein DRAL/FHL2 Binds to the Cytoplasmic Domain of Several α and β Integrin Chains and Is Recruited to Adhesion Complexes. J. Biol. Chem. 275, 33669–33678. 10.1074/jbc.m002519200 10906324

[B110] Wozniak M. A. Modzelewska K. Kwong L. Keely P. J. (2004). Focal Adhesion Regulation of Cell Behavior. Biochim. Biophys. Acta (Bba) - Mol. Cel Res. 1692, 103–119. 10.1016/j.bbamcr.2004.04.007 15246682

[B111] Wu P. H. Opadele A. E. Onodera Y. Nam J. M. (2019). Targeting Integrins in Cancer Nanomedicine: Applications in Cancer Diagnosis and Therapy. Cancers (Basel) 11, 1783. 10.3390/cancers11111783 PMC6895796 31766201

[B112] Wu R. Y. Gill G. N. (1994). LIM Domain Recognition of a Tyrosine-Containing Tight Turn. J. Biol. Chem. 269, 25085–25090. 10.1016/s0021-9258(17)31502-8 7929196

[B113] Yao M. Goult B. T. Klapholz B. Hu X. Toseland C. P. Guo Y. (2016). The Mechanical Response of Talin. Nat. Commun. 7, 11966. 10.1038/ncomms11966 27384267 PMC4941051

[B114] Ye F. Kim C. Ginsberg M. H. (2012). Reconstruction of Integrin Activation. Blood 119, 26–33. 10.1182/blood-2011-04-292128 21921044 PMC3251231

[B115] Yu C.-h. Luo W. Sheetz M. P. (2012). Spatial-temporal Reorganization of Activated Integrins. Cell Adhes. Migration 6, 280–284. 10.4161/cam.20753 PMC3427242 22863737

[B116] Zacharchenko T. Qian X. Goult B. T. Jethwa D. Almeida T. B. Ballestrem C. (2016). LD Motif Recognition by Talin: Structure of the Talin-DLC1 Complex. Structure 24, 1130–1141. 10.1016/j.str.2016.04.016 27265849 PMC4938799

[B117] Zaidel-Bar R. Itzkovitz S. Ma'ayan A. Iyengar R. Geiger B. (2007a). Functional Atlas of the Integrin Adhesome. Nat. Cel Biol 9, 858–867. 10.1038/ncb0807-858 PMC2735470 17671451

[B118] Zaidel-Bar R. Milo R. Kam Z. Geiger B. (2007b). A Paxillin Tyrosine Phosphorylation Switch Regulates the Assembly and Form of Cell-Matrix Adhesions. J. Cel Sci 120, 137–148. 10.1242/jcs.03314 17164291

[B119] Zhang P. Azizi L. Kukkurainen S. Gao T. Baikoghli M. Jacquier M. C. (2020). Crystal Structure of the FERM-Folded Talin Head Reveals the Determinants for Integrin Binding. Proc. Natl. Acad. Sci. U S A. 117 (51), 32402–32412. 10.1073/pnas.2014583117 33288722 PMC7768682

[B120] Zhang X. Jiang G. Cai Y. Monkley S. J. Critchley D. R. Sheetz M. P. (2008a). Talin Depletion Reveals independence of Initial Cell Spreading from Integrin Activation and Traction. Nat. Cel Biol 10, 1062–1068. 10.1038/ncb1765 PMC2746969 19160486

[B121] Zhang Z. M. Simmerman J. A. Guibao C. D. Zheng J. J. (2008b). GIT1 Paxillin-Binding Domain Is a Four-helix Bundle, and it Binds to Both Paxillin LD2 and LD4 Motifs. J. Biol. Chem. 283, 18685–18693. 10.1074/jbc.m801274200 18448431 PMC2441563

[B122] Zhu C. Kong Z. Wang B. Cheng W. Wu A. Meng X. (2019a). ITGB3/CD61: a Hub Modulator and Target in the Tumor Microenvironment. Am. J. Transl Res. 11, 7195–7208. 31934272 PMC6943458

[B123] Zhu J. Zhou Q. Xia Y. Lin L. Li J. Peng M. (2020). GIT/PIX Condensates Are Modular and Ideal for Distinct Compartmentalized Cell Signaling. Mol. Cel 79, 782–796. 10.1016/j.molcel.2020.07.004 32780989

[B124] Zhu L. Liu H. Lu F. Yang J. Byzova T. V. Qin J. (2019b). Structural Basis of Paxillin Recruitment by Kindlin-2 in Regulating Cell Adhesion. Structure 27, 1686–1697. 10.1016/j.str.2019.09.006 31590942 PMC6894617

